# HIV-1 Tat exacerbates lipopolysaccharide-induced cytokine release via TLR4 signaling in the enteric nervous system

**DOI:** 10.1038/srep31203

**Published:** 2016-08-05

**Authors:** Joy Guedia, Paola Brun, Sukhada Bhave, Sylvia Fitting, Minho Kang, William L. Dewey, Kurt F. Hauser, Hamid I. Akbarali

**Affiliations:** 1 Department of Pharmacology and Toxicology, Virginia Commonwealth University, Richmond, VA 23298, USA; 2 Department of Molecular Medicine, University of Padova, Padova, Italy

## Abstract

The loss of gut epithelium integrity leads to translocation of microbes and microbial products resulting in immune activation and drives systemic inflammation in acquired immunodeficiency syndrome (AIDS) patients. Although viral loads in HIV patients are significantly reduced in the post-cART era, inflammation and immune activation persist and can lead to morbidity. Here, we determined the interactive effects of the viral protein HIV-1 Tat and lipopolysaccharide (LPS) on enteric neurons and glia. Bacterial translocation was significantly enhanced in Tat-expressing (Tat+) mice. Exposure to HIV-1 Tat in combination with LPS enhanced the expression and release of the pro-inflammatory cytokines IL-6, IL-1β and TNF-α in the ilea of Tat+ mice and by enteric glia. This coincided with enhanced NF-κB activation in enteric glia that was abrogated in glia from TLR4 knockout mice and by knockdown (siRNA) of MyD88 siRNA in wild type glia. The synergistic effects of Tat and LPS resulted in a reduced rate of colonic propulsion in Tat+ mice treated with LPS. These results show that HIV-1 Tat interacts with the TLR4 receptor to enhance the pro-inflammatory effects of LPS leading to gastrointestinal dysmotility and enhanced immune activation.

HIV infection is characterized by intestinal mucosal damage leading to increased translocation of bacteria and viruses into the gut wall thereby exposing other cells and organs to bacterial and viral proteins and predisposing to systemic immune activation^1^,^2^,^3^,^4^. Clinical and experimental data also suggest that increased translocation correlates with progression to AIDS^5^,^6^. Several studies have suggested that microorganisms and microbial products from the lumen of the gut are responsible for this immune activation and inflammation^3^,^6^. Lipopolysaccharide (LPS), a major component of the cell membrane of Gram-negative bacteria, is generally used as a tool to quantify the rate of bacterial translocation in HIV and serum LPS binding protein is significantly reduced in HIV infected individuals^7^ indicating increased leakiness of the gut epithelial barrier. LPS is recognized by toll like receptor 4 (TLR4) and triggers the secretion of pro-inflammatory cytokines and can additionally regulate intestinal homeostasis^8^,^9^.

In the post-cART era, the pathologic consequences of HIV continue despite diminished viral loads, suggesting that neuroinflammation and tissue injury may result from viral products including the transactivator of transcription (HIV-1 Tat)^10^,^11^,^12^, independent of viral replication. There is no evidence of direct neuronal infection by HIV. Studies in the brain show that viral toxins such as Tat, a 14 kDa viral protein, responsible for regulation of transcription, and released by intact infected cells are able to interact and modulate neuronal function^13^,^14^. We have recently shown that Tat profoundly affects the excitability of myenteric neurons, increases inflammatory mediators in the myenteric plexus and alters gastrointestinal motility^15^. Furthermore, Tat also sensitized enteric neurons to morphine^16^. In this study, we sought to determine the interactive effects of Tat and LPS on enteric neurons and glia, and examine its role in inflammation and GI disturbances, that are commonly observed in HIV-infected patients.

## Results

### Bacterial translocation in Tat transgenic mice

**Figure 1:**
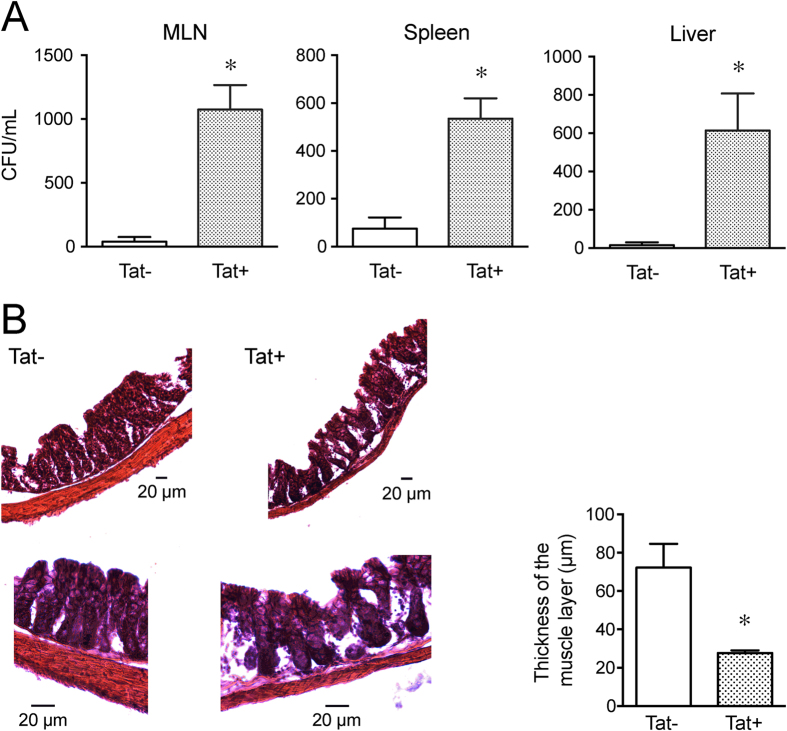
Bacterial Translocation in Tat transgenic mice. Quantification of bacteria colony forming units in Mesenteric lymph node, Spleen, Liver from Tat+ and Tat− transgenic mice. Data plotted as Mean ± SEM. n = 3. P < 0.05 (Students *t test*) *Vs Tat− (**A**). Hematoxylin and Eosin staining of Tat+ and Tat− transgenic mice colon. The muscle wall thickness was measured across several areas on each section and averaged for each colon (**B**). The bar graph is the average for each colon (n = 4).

The effect of Tat on gut bacterial translocation was determined by counting the number of colony forming units of bacteria per mL of homogenates of mesenteric lymph nodes (MLN), liver and spleen in Tat transgenic mice. Tat expression in the doxycycline (DOX)-inducible, HIV-Tat_1–86_ transgenic mice is under the control of the tetracycline responsive, glial fibrillary acidic protein (GFAP) – selective promoter. We have previously shown that Tat mRNA is highly expressed in the mouse ileum longitudinal muscle myenteric plexus preparation indicating that GFAP(+) cells in myenteric plexus also express Tat upon induction^15^. Tat+ and Tat− transgenic mice were administered DOX to induce the expression of Tat as described previously^15^. The DOX diet was replaced by regular chow for 2 weeks without DOX to allow for recolonization of gut microbiota and MLN, spleen and liver were aseptically collected, weighed, homogenized and cultured. The MLN, spleen and liver of Tat+ mice had significantly higher colony forming units (CFU)/mL of bacteria in tissue homogenates than Tat− mice (Fig. 1A). The highest rate of translocation was to the MLN. Histological examination of the Tat+ mice showed significant disruption of the epithelium and a decrease in the smooth muscle layer thickness compared to Tat− mice (Fig. 1B).

### Ilea of Tat-expressing (Tat+) mice were more sensitive to LPS induced increase in pro-inflammatory cytokines than Tat**−** mice

**Figure 2:**
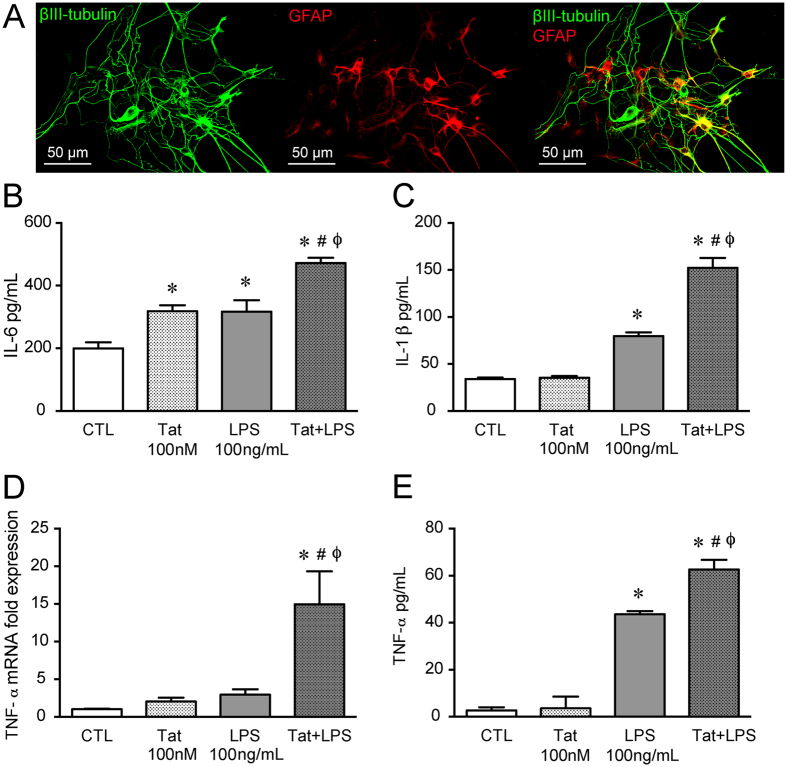
Tat and LPS interaction in neuron-glia co-cultures and LMMP from adult mouse ileum. (**A**) Representative confocal microscopic images of enteric β-III tubulin (green) and glial fibrillary protein (GFAP- red) showing the presence of neurons and glia isolated from the adult mouse LMMP. (**B**) IL-6 release from isolated mouse neuron/glia co-culture treated with 100 nM Tat, 100 ng/ml LPS or Tat+ LPS for 16 h measured by ELISA. (**C**) IL-1β release from neuron/glia co-culture treated with 100 nM Tat, 100 ng/ml LPS and Tat+ LPS for 16 h measured by ELISA (**D**) mRNA expression of TNF-α of mouse LMMP treated with 100 nM Tat, 100 ng/ml LPS and Tat+ LPS for 16 h. (**E**) TNF-α release from mouse LMMP treated with 100 nM Tat, 100 ng/ml LPS or Tat+ LPS for 16 h measured by ELISA. In each experiment the n = 3 and p < 0.05. One-way ANOVA plus Bonferroni’s post hoc test. *Vs CTL, #Vs Tat, ϕVs LPS.

We have previously shown that pro-inflammatory cytokines, IL-6 and RANTES, are upregulated in Tat+ mice ilea as well as upon exposure to exogenous Tat in isolated enteric neurons/glia co-culture^15^. To assess the *in vitro* effect of Tat and LPS combination on pro-inflammatory cytokine expression and release, we used; a) primary cultures of neurons and glia isolated from the adult mouse ileum (Fig. 2A) b) whole longitudinal muscle myenteric plexus (LMMP) preparations, and c) the CRL-2690 enteric glia cell line.

The release of pro-inflammatory cytokines in the neuron/glia co-culture following treatments with either Tat (100 nM) alone, LPS (100 ng/mL) alone, or the two in combination for 16 h was determined. The Tat concentration (100 nM) was chosen from a range reported to elicit functional deficits in glia and neurons in the CNS and similar to those occurring in HIV-1^17^,^18^,^19^. We have previously determined the specificity of Tat following heat inactivation^15^ where heat-inactivated Tat did not elicit enhanced excitability of enteric neurons. Both Tat and LPS increased IL-6 release and showed further enhanced response when combined (Tat+ LPS) (Fig. 2B). Similarly, the Tat/LPS interaction was also observed for IL-1β release whereby Tat alone did not increase IL-1β but the combination resulted in significant enhancement (Fig. 2C). In the mouse-ileum LMMP preparation, similar to previous reports in enteric neuronal/glia co-culture^15^, Tat alone did not increase TNF-α mRNA or protein secretion (Fig. 2D,E), however, in combination with LPS (100 ng/ml) there was upregulation of both mRNA and cytokine release following 16 h treatment. While LPS (100 ng/ml) did not increase mRNA expression, it significantly increased TNF-α release in the LMMP preparation.

**Figure 3:**
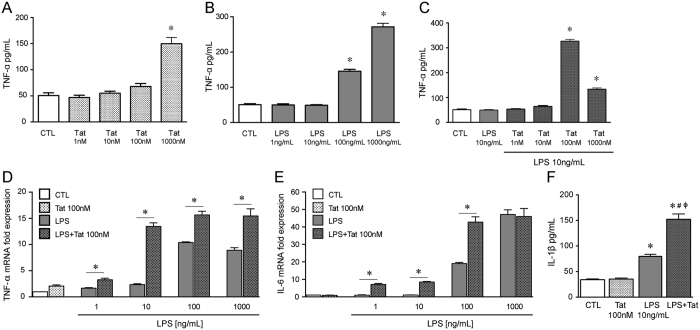
Tat and LPS interaction in the CRL-2690 enteric glia cell line. (**A**) Release of TNF-α of CRL-2690 enteric glia after treatment with 1–1000 nM Tat for 16 h. (**B**) Release of TNF-α of CRL-2690 enteric glia after treatment with 1–1000 ng/mL LPS for 16 h. (**C**) Release of TNF-α of CRL-2690 enteric glia after treatment with 10 ng/mL LPS or LPS + 1–1000 nM Tat for 16 h. (**D**) Normalized fold TNF-α mRNA expression of CRL-2690 enteric glia after treatment with 100 nM Tat in combination with 1, 10, 100 or 1000 ng/mL LPS for 16 h. (**E**) Normalized fold IL-6 mRNA expression of CRL-2690 enteric glia after treatment with 100 nM Tat in combination with 1, 10, 100 or 1000 ng/mL of LPS for 16 h. (**F**) Release of IL-1β of CRL-2690 enteric glia after treatment with 100 nM Tat or 10 ng/mL LPS or both for 16 h. For each study n = 3, P < 0.05. One-way ANOVA plus Bonferroni’s post hoc test. *Vs CTL, #Vs Tat, ϕVs LPS.

To further determine the role of enteric glia in Tat/LPS- mediated increase in pro-inflammatory cytokines, we assessed the effects of Tat and LPS on the CRL-2690 enteric glial cell line. We initially determined the dose-response for Tat and LPS on TNF-α release (Fig. 3A,B). When 100 nM Tat was examined against 10 ng/ml LPS, concentrations at which neither induced significant TNF-α release, the combination resulted in enhanced TNF-α release and mRNA expression (Fig. 3C,D). Similarly, the synergistic effects of Tat and LPS were also observed for IL-6 mRNA and IL-1β release (Fig. 3E,F).

**Figure 4:**
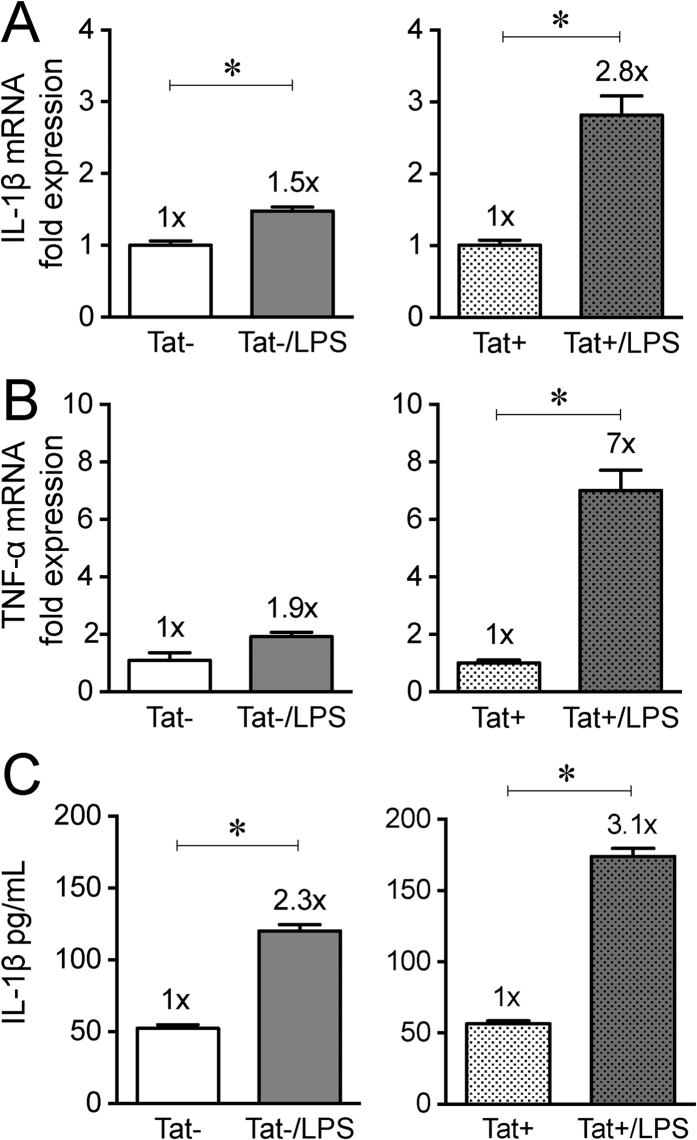
Cytokine expression and release in Tat mice following LPS treatment *in vivo*. Normalized fold mRNA expression of Il-1β (**A**) and TNF-α (**B**) of ileum of Tat transgenic mice administered with LPS at a dose of 50 μg/mL in drinking water for 1 week. Tat+ mice were more sensitive to LPS induced increase in cytokine expression. (**C**) IL-1β protein measured by ELISA. n = 5–6. P < 0.05 (Student’s *t test*) *Vs Tat− or Tat+.

To further examine the possible interaction of LPS with Tat *in-vivo*, LPS (50 μg/ml) was administered in drinking water for one week to Tat+ and Tat− mice. Expression of both IL-1β and TNF-α was significantly enhanced in the ileum of Tat+ mice treated with LPS. In the ilea of Tat + mice, LPS produced a 2.8-fold increase in IL-1β mRNA compared to a 1.5 fold increase observed in Tat− mice (Fig. 4A). Also, LPS produced a 7-fold increase in TNF-α mRNA, which was significantly higher than the 1.9-fold increase in Tat− observed in the ilea (Fig. 4B). LPS induced a higher increase in IL-1β protein levels in the ilea of Tat+ (3.1-fold increase) than in Tat− mice (2.3-fold increase) (Fig. 4C).

### Tat LPS interaction is TLR4 mediated

**Figure 5:**
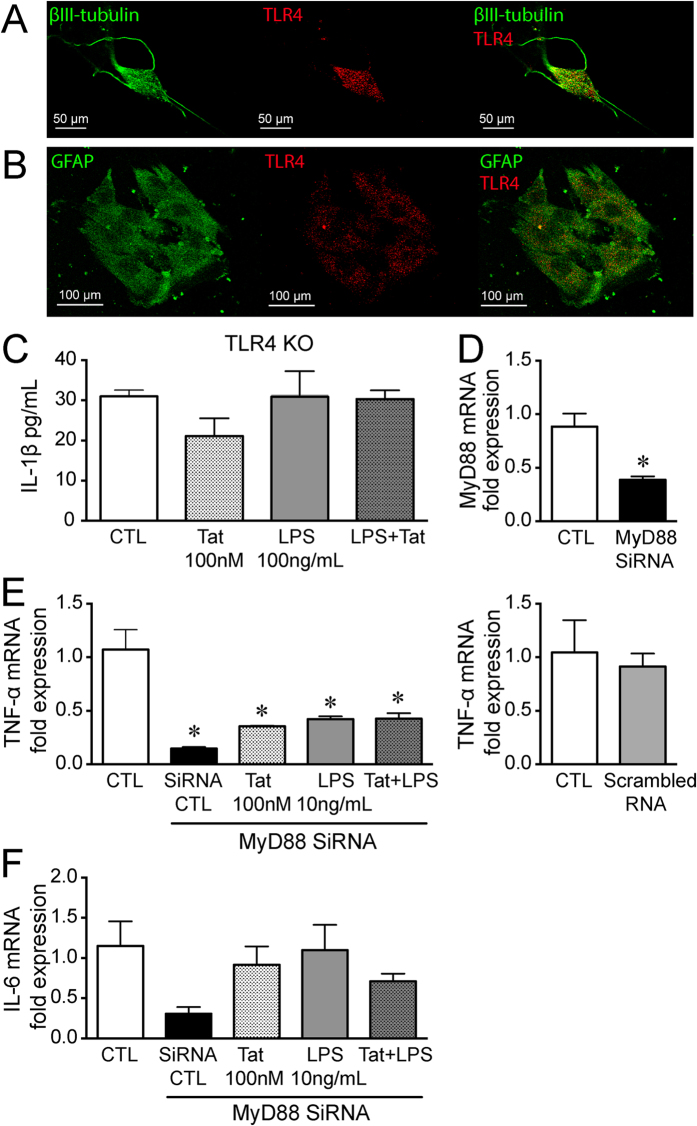
Tat LPS interaction is TLR4 mediated. (**A**) Representative confocal microscopic images of enteric β-III tubulin (green) and TLR4 (red) showing the expression of TLR4 on neurons. (**B**) Representative confocal microscopic images of enteric GFAP (green) and TLR4 (red) showing the expression of TLR4 on enteric glia. (**C**) Release of IL-1β in neuron-glia co-cultures isolated from TLR4 knockout mice after treatment with 100 nM Tat, 10 ng/mL LPS or Tat+ LPS for 16 h. (**D**) RT-PCR showing knock down of MyD88 in CRL-2690 enteric glia. (**E**) Normalized fold mRNA expression of TNF-α and IL-6 (**F**) in CRL-2690 enteric glia following knock-down of MyD88 or with scrambled RNA and treated with 100 nM Tat, 10 ng/ml LPS or Tat+ LPS. For each set of experiments N = 3, p < 0.05. *vs CTL.

Both enteric neurons and glia express TLR4 (Fig. 5A,B). To determine the involvement of TLR4 in this enhanced response to LPS by Tat, primary neuronal/glial cultures were isolated from TLR4 knockout mice. The primary cultures were treated with Tat and LPS for 16 h. Neither 100 nM Tat nor 100 ng/mL LPS alone or in combination (Tat+ LPS) increased IL-1β levels (Fig. 5C) establishing the role of TLR4 in Tat-LPS interactions in the gut.

We then determined the role of MyD88, an adaptor protein that mediates TLR4 signaling. Interestingly, in glial cells treated with siRNA for MyD88 (Fig. 5D), the expression of TNF-α was significantly reduced in basal conditions. The effects of both Tat and LPS were blocked in cells with MyD88 siRNA (Fig. 5E). The knockdown of MyD88 also prevented the sensitizing effects of combining Tat and LPS (Fig. 5E,F).

**Figure 6:**
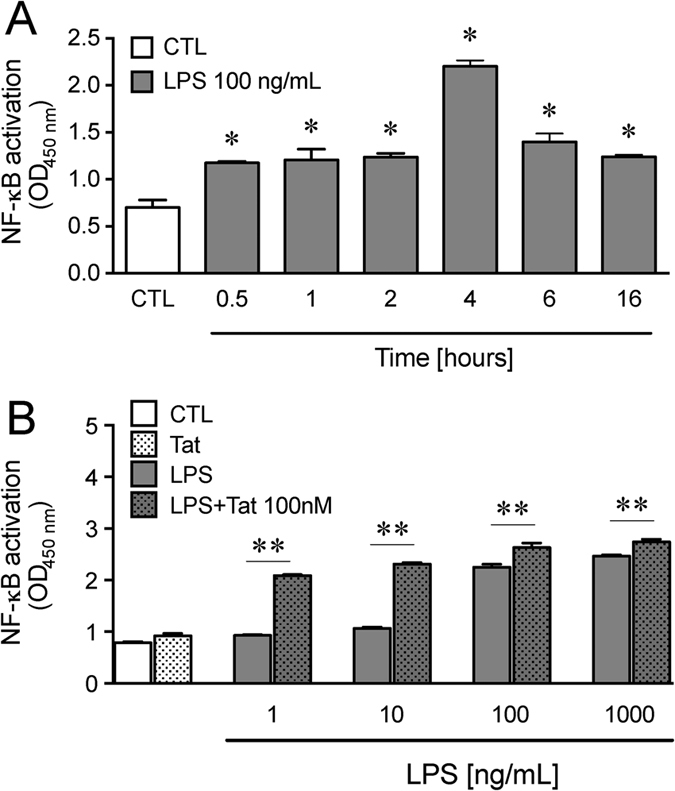
NF-κB activation. (**A**) Time course of NF-κB activation in CRL-2690 enteric glia treated with 100 ng/mL LPS measured as the amount of translocation of NF-κB in the nucleus. n = 3, P < 0.05 (Student’s *t test*) *Vs CTL. (**B**) NF-κB activation in CRL-2690 enteric glia treated with 100 nM Tat and/or (1, 10,100, 1000) ng/mL LPS with Tat for 4 h. n = 3, P < 0.05 (two-way ANOVA) ** Vs LPS.

The sensitization between Tat and LPS was also observed on NF-κB activation. LPS time dependently activates NF-κB with the highest amount of activation occurring at 4 h following continuous LPS treatment (Fig. 6A). Despite findings that 100 nM Tat did not increase NF-κB activation, low concentrations of LPS significantly sensitized NF-κB activation to Tat exposure (Fig. 6B).

### Tat+ mice were more sensitive to LPS mediated decrease in colonic motility than Tat**−** mice

To further examine the functional aspects of the Tat/LPS interaction on GI motility, a video based gastrointestinal motility monitor (GIMM) system was used to generate spatiotemporal maps of the ileum and assess pellet expulsion from the colon.

**Figure 7:**
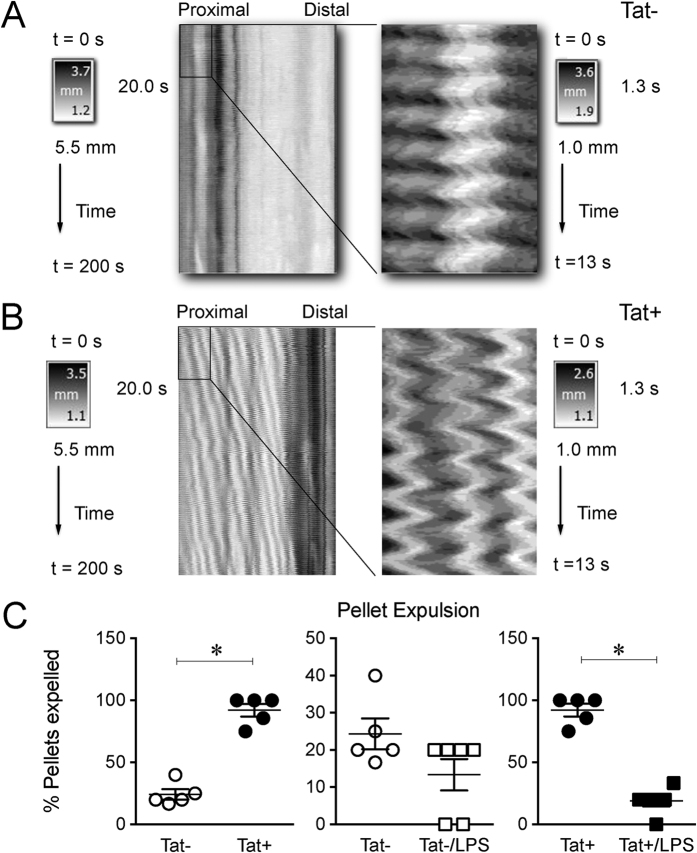
Tat+ mice were more sensitive to LPS mediated decrease in colonic motility than Tat− mice. (**A**) Representative spatiotemporal maps of the mouse ileum with the proximal portion zoomed-in in Tat−. (**B**) Representative spatiotemporal maps of the mouse ileum with the proximal portion zoomed-in, in Tat+ mice. LPS was administered at a dose of 50 μg/mL, in drinking water for 1 week. (**C**) Quantification of colonic transit measured as rate of expulsion of natural pellets in 30 min. n = 5–6. P < 0.05 (Students *t test*) *Vs Tat− or Tat+.

Figure 7A,B show a typical recording of the spatiotemporal map of ileal contractions from Tat− and Tat+ mice. The spatiotemporal maps revealed significant high frequency, low amplitude contractions throughout the ileum (Supplemental Fig. 1). These contractions occurred across the entire length of the ileum at varying frequencies. The contractions, which we denoted as “ripples”, are similar to the findings in gastrointestinal tissue from embryonic rats reported by Roberts *et al*. (19) and were greater in the proximal ileum.

The interaction between LPS and Tat on colonic propulsion was determined by examining the expulsion of natural colonic pellets in isolated colon segments. Colonic propulsion was recorded for 30 min and the data presented as percent colonic pellet expulsion rate (supplementary videos). There was no difference in the number of pellets between the two groups when removed from the animal (Supplementary Table 2). Tat− mice expelled 24.3% of their natural pellets, whereas Tat+ mice expelled 92.1% of the natural pellets. Interestingly, colonic propulsion from the LPS treated mice had reduced pellet expulsion rates: 13.3% in Tat− mice and 18.8% in Tat+ mice (Fig. 7C).

## Discussion

Microbial translocation in HIV patients is now recognized as an important contributor of HIV pathogenesis. In the present study, we demonstrate that Tat sensitizes the gut to LPS-mediated pro-inflammatory cytokine increases and that this increase in inflammation significantly disrupts gastrointestinal motility. This interaction appears to be mediated via TLR4. Our findings point to a novel interaction between viral proteins and bacterial lipopolysaccharides in the enteric nervous system.

The ENS regulates various motor patterns in the gut. Two main motor patterns are observed in the small intestine. These include (i) peristalsis, which is responsible for propelling the bolus and (ii) segmented contractions, which mixes the bolus allowing time for digestion and absorption of nutrients^20^. These motility patterns have been shown to be controlled by interaction of the ENS, interstitial cells of Cajal (ICCs) and smooth muscle^20^,^21^. Gut inflammation has been shown to cause remodeling of the enteric nervous system in various GI diseases^22^. We hypothesized that the increase in cytokine release observed will remodel cells of the ENS and thus affect GI motility patterns. We have previously reported that myenteric neurons from the ileum of Tat+ transgenic mice are significantly more excitable, and cecal water content is markedly increased^15^. Consistent with this, the spatiotemporal maps of the ileum showed enhanced activity in the Tat+ mice. While the colonic propulsion was also increased in the Tat+ mice, interestingly LPS provided in the drinking water reduced fecal pellet propulsion. It is unclear if this paradoxical effect of LPS results from exaggerated levels of inflammation beyond that seen in mice following Tat induction alone. It is noteworthy that the expression of both IL-1β and TNF-α were exaggerated in Tat+ mice treated with LPS.

Inflammation is known to directly correlate with the rate of HIV replication and a heightened progression to AIDS^23^,^24^. GI inflammation is largely regulated by the cells of the ENS. Enteric neurons and glia have been shown to undergo structural and functional remodeling during GI inflammation^25^,^26^,^27^. Previously we showed that Tat directly modulates the function of enteric neuronal-glial co-cultures leading to an increase in pro-inflammatory cytokine release^15^. This was confirmed in the present study whereby Tat increased IL-6 but not TNF-α. Interestingly, although Tat did not increase IL-1β expression in the isolated neurons or glia, we previously found that IL-1β expression is enhanced in the ileum of Tat+ mice. This is consistent with the thesis that bacterial translocation increases LPS levels and interactions with ENS cells resulting in enhanced inflammatory responses.

Enteric glia are a major component of the myenteric plexus. They have been implicated in the regulation of gut epithelial membrane integrity^28^,^29^, mucosal defense against gut bacteria^30^ and GI inflammation^31^,^32^. Enhanced microbial translocation observed in Tat+ mice may allow for potential increase in the inflammatory cytokines by stimulating cells that were not previously exposed to viral and bacterial products. The enhanced release of several pro-inflammatory cytokines by a combination of Tat and LPS in isolated myenteric neuron/glia co-cultures was confirmed in an enteric glial cell line and in the ilea of the Tat+ transgenic mice.

Since our goal was to elucidate the interaction between LPS and the HIV Tat protein in the intestinal wall, LPS was given in drinking water to mimic the physiological exposure of cells in the intestinal wall to bacterial LPS. Humans are constitutively exposed to LPS throughout their lives. Several studies report that almost all foods contain 1 ng to 1 μg of LPS per gram. Moreover, humans are constantly exposed to huge amounts of bacteria in oral and intestinal mucosa. The estimated number of human commensal bacteria range from 10 ^ 3 to 10 ^ 12 per gram of tissue. In healthy subjects, the continuous exposure to LPS may be important for the maintenance of host immune balance and protection from bacterial infections in the intestine^33^,^34^. However, the systemic and local (gut) clinical significance of this LPS exposure in HIV patients, suffering from a debilitated immune system, is still a matter of debate. Furthermore, oral administration of LPS demonstrates different consequences when compared to intravenous or intraperitoneal administration. Systemic administration of LPS results in activation of macrophages and massive systemic pro-inflammatory cytokines secretion. In humans and animals, endotoxemia causes fever, hypotension and also gut barrier failure, further increasing bacterial LPS translocation^35^,^36^. In transgenic GFAP-luc animal model, systemic administration of LPS induces astrocyte activation^37^. The *in vivo* systemic LPS administration on activation of cells of the enteric nervous system are not known. On the other hand, oral administration of LPS results in its translocation across the gut wall and its penetration into lymphoid tissues, such as Peyer’s patches and the mesenteric lymph nodes causing the localized activation of specific immune compartments, with no evidence of toxicity or alterations in the structure of the small intestine^38^. Thus, oral administration of LPS was employed to best mimic the route by which intestinal immune cells would be normally exposed to LPS or other PAMPs originating from the microbiome [or LPS]. Alternatively, Tat protein is not present in HIV virions and is only produced after a cell is infected. Therefore, in the gut wall, Tat would normally be produced by infected T-cells, macrophages and enteric glia. Our experiments were designed to mimic the route of exposure to LPS and HIV Tat to attempt to accurately trigger the shared signaling pathways involved in the interaction of bacterial and viral products. A significant finding from this study was the enhanced expression of the pro-inflammatory cytokines in the Tat+ mice when LPS was administered orally.

TLR4 is the main receptor for LPS. When LPS binds to TLR4, a MyD88 and/or TRIF dependent pathway is activated^8^. Activation of the MyD88 dependent pathway leads to downstream activation of NF-κB, which drives the synthesis of pro-inflammatory cytokines such as TNF-α, IL-6 and IL-1β. Moreover, Tat has recently been shown to bind to the TLR4 receptor and to induce cytokine release in a TLR4 dependent manner^39^. The enhancement in inflammatory cytokine levels observed after treating enteric neurons and/or glia with a combination of Tat and LPS in wild type mice was absent in TLR4 knockout mice, and when MyD88 was knocked down by siRNA. LPS-induced activation of NF-κB was concentration-dependent. When treated in combination with escalating concentrations of Tat, LPS activated NF-κB to a significantly higher level with each concentration increase. These data suggest that the interaction between Tat and LPS in the enteric nervous system likely involves TLR4. A possible mechanism may involve the allosteric modulation of TLR4 by Tat, thus enhancing the effect of LPS.

Loss of gut epithelial integrity, gut bacterial translocation and immune activation are hallmarks of HIV disease. We show here that the interaction of Tat and LPS causes enteric neurons and/or glia to release significantly higher amounts of pro-inflammatory cytokines. This sensitization effect is a TLR4-mediated process occurring via a MyD88-dependent pathway. Increases in pro-inflammatory cytokines have been shown to further affect gut epithelial membrane integrity. This may lead to further enhancement of bacterial translocation. Since patients who are on cART have reduced rates of viral replication, it is believed that the ongoing inflammation and immune activation may be primarily due to bacterial translocation. This persistent inflammation in these patients may lead to further complications such as atherosclerosis, neurodegenerative disorders and other end organ complications. Similar to the findings reported here in the enteric nervous system, recent studies show that HIV-infected primary astrocytes also respond with greater sensitivity to LPS^40^.

## Materials and Methods

All experiments were conducted in accordance with the procedures reviewed and approved by the Institutional Animal Care and Use Committee (IACUC) at Virginia Commonwealth University.

### Isolation and culture of neurons from the adult mouse myenteric plexus

Myenteric neurons and glia were isolated as described recently^41^. Briefly, after euthanizing mice, the ileum was immediately removed and placed in ice-cold Krebs solution (118 mM NaCl, 4.6 mM KCl, 1.3 mM NaH_2_PO_4_, 1.2 mM MgSO_4_, 25 mM NaHCO_3_, 11 mM glucose and 2.5 mM CaCl_2_) bubbled with carbogen (95% O_2_/5% CO_2_). The ileum contents were discarded by passing Krebs solution using a syringe. The ileum was then divided into short segments which were threaded longitudinally on a plastic rod through the lumen and the longitudinal muscle containing the myenteric plexus (LMMP) strips were obtained using a cotton-tipped applicator. LMMP strips were rinsed three times in 1 ml Krebs and gathered by centrifugation (350 × *g*, 30 sec). LMMP strips were then minced with scissors and digested in 1.3 mg/ml collagenase type II (Worthington) and 0.3 mg/ml bovine serum albumin in bubbled Krebs (37 °C) for 1 h, followed by 0.05% trypsin for 7 min. Following each digestion, cells were triturated and collected by centrifuge (350 x *g* for 8 min). Cells were then plated on laminin (BD Biosciences) and poly-D-lysine coated coverslips in Neurobasal A media containing B-27 supplement, 1% fetal bovine serum, 10 ng/ml glial cell line-derived neurotrophic factor (GDNF, Neuromics, Edina, MN), and penicillin/streptomycin. Half of the cell media was replaced every 2–3 days with fresh complete neuron media.

All chemicals and reagents were obtained from Sigma Aldrich (St Louis, MO), unless otherwise noted, except cell culture reagents, which were purchased from Gibco (Grand Island, NY) and the HIV Tat_1–86_ protein (Immunodiagnostics, Inc.). Male Swiss Webster mice (25–30 g, Harlan Sprague Dawley, Inc.) or male and female doxycycline (DOX)-inducible, HIV-Tat_1–86_ transgenic mice (25–30 g) were used. The HIV-Tat_1–86_ transgenic mouse model was developed on a C57BL/6J hybrid background and is described in detail elsewhere^42^ (17). Tat expression, which is under the control of a tetracycline responsive, glial fibrillary acidic protein (GFAP)-selective promoter, was induced with a specially formulated chow containing 6 mg/g DOX (Product#: TD.09282; Harlan, Indianapolis, IN, USA), fed to both the Tat− controls and the inducible Tat+ mice. Toll like receptor 4 (TLR4) knockout mice were used to determine the role of TLR4 in the interaction of LPS and Tat. Mice were euthanized by cervical dislocation.

### LPS Administration

To study the interaction of Tat and LPS *in vivo*, Tat transgenic (Tat+ and Tat−) mice were administered DOX for 2 weeks to induce the expression of Tat followed by a period of 2 weeks without DOX to allow for recolonization of gut microbiota. LPS was administered at a dose of 50 μg/mL, in drinking water for 1 week^43^.

### Cytokine release

Cytokine release was measured by Enzyme-linked immunosorbent assay (ELISA) kits (Bioscience, an Affymetrix company, San Diego, USA). Supernatants were obtained from mouse primary neuron/glia cultures or from the enteric glial cells (CRL-2690 *Rattus rattus*) after treatment with Tat, LPS, or a combination of both at the indicated time points and concentrations. Ileal samples and LMMP preparations obtained from Tat+ and Tat− mice with or without LPS treatment were homogenized in 0.2 parts of PBS containing protease inhibitors (Roche Life Science). Following centrifugation (13,000 rpm, 10 min at 4 °C) supernatants were collected and assessed for cytokine content.

### RT-PCR

RNA was extracted from the enteric glial cell line (CRL-2690) using RNAqueous® Total RNA Isolation Kit (life technologies) or from mouse ileum or the LMMP using TRIZOL (Invitrogen, Carlsbad, CA, USA) following manufacturer’s protocol. It was then quantified using spectrophotometer (BIORAD Smart Spec Plus). cDNA synthesis and subsequent polymerization was performed in a one-step using the iTaq Universal SYBR Green One-Step Kit (Bio-Rad, Hercules, CA, USA). The reaction mixture (20 μl) contained 200 nM forward primer, 200 nM reverse primer, 1× iTaq universal SyBR Green reaction mix, 1× iScript reverse transcriptase, and 200 ng total RNA. Real time PCR was performed using a MiniOpticon Real-Time PCR System (Bio-Rad, Hercules, CA, USA). Specific primers for IL-6, TNF-α, and IL-1β, Na_v_1.7 and Na_v_1.8 were used. 18 s RNA was used as the internal control. Experiments were performed in triplicates from three separate samples. The mean normalized fold expression ± SEM are plotted. The primers used are included in Supplementary Table 1.

### MyD88 siRNA knock down

To determine the role of MyD88 on Tat and LPS interactions, MyD88 siRNA (MyD88 siRNA (r): sc-106986) was used to knock down MyD88 in the enteric glial cell line (CRL-2690) following the manufacturers protocol. Briefly, 2 × 10^5^ cells were seeded in 6 well plates for 18–24 h. At 60–80% confluence, cells were transfected with 2–8 μl of the siRNA duplex for 6 h after which 1 mL of culture media containing 2 times FBS and penicillin/streptomycin was added and cultured for 18–24 h. The transfection media was replaced with fresh culture media and cultured for 24 h. MyD88 knockdown was confirmed by RT-PCR using specific MyD88 primers. The effects of LPS and Tat on TNF-α and IL-6 were determined using RT-PCR.

### NF-κB assay

To determine the role of NF-κB on Tat and LPS interactions, CRL-2690 cells were treated with Tat and/or LPS for the indicated times (0.5, 1, 2, 4, 6, 16) h. When NF-κB is activated, it translocates to the nucleus where it drives gene expression. The nuclear portions were extracted and nuclear/activated NF-κB p65 transcript was quantified using NF-κB p65 binding ELISA kit (Abcam). The amount of nuclear NF-κB represents the amount of activated NF-κB.

### Immunocytochemistry

Isolated neurons and glia cells cultured for 4 days to 2 weeks on coverslips were fixed in 4% formaldehyde for 30 min. Cells were permeabilized with 0.01% Triton X-100 in PBS (30 min), and blocked with 10% goat serum (1 h). Preparations were incubated with the primary antibody overnight at 4 °C. Primary antibodies used were as follows: neuronal specific anti-βIII-tubulin (rabbit, Abcam ab18207-100, 1:500), anti-GFAP (mouse, Santa Cruz, 1:500), anti-βIII-tubulin (mouse, Abcam ab7751, 1:500), and TLR4 (rabbit, Santa Cruz, 1:200). Following 3 washes in PBS, cells were incubated with the appropriate secondary antibodies; goat anti-rabbit Alexa 488 Dye (Molecular Probes, 1:1,000, 1 h, RT) and goat anti-mouse Alexa 594 Dye (Molecular Probes, 1:1,000, 1 h, RT). Visualization was performed on an Olympus Fluoview Confocal Microscope and software (v5.0).

### Hematoxylin and eosin (H&E) staining

Tat transgenic mice were dissected, the colon was isolated and immediately placed in ice-cold Krebs solution continuously bubbled with Carbogen gas (95% O_2_/5% CO_2_). The fecal pellets were gently flushed using 10 ml syringes attached to a blunted needle. The tissue was then embedded in an Optimal Cutting Temperature compound (OCT, Sakura Finetek, Torrance, CA). 20-μm cryosections were cut and fixed with 4% paraformaldehyde in PBS. The sections were then stained following standard hematoxylin and eosin (H&E, Sigma-Aldrich, St. Louis, MO) staining protocol.

### Gastrointestinal Motility Monitor (GIMM)

Colonic motility was measured using the gastrointestinal motility monitor (Catamount Research and Development, Inc., Vermont, USA). We slightly modified Barnes *et al*.’s methodology^44^. After euthanizing adult Tat transgenic mice, their colons were excised immediately and placed in an illuminated organ bath, continuously perfused with Krebs solution at 35.5 ± 0.5 °C. The Krebs solution was constantly bubbled with carbogen gas (95% O_2_/5% CO_2_). The entire colon containing natural pellets was anchored with pins at the mesenteries of the anal and oral ends to prevent interfering with pellet movement and over-manipulation of the tissue. The remaining mesenteries were gently trimmed. Typically, it took ≤5 min from excising the colon until when the recording was started. The number of pellets expelled in 30 min was recorded and was used to determine the rate of colonic transit. The ileum was also excised starting immediately proximal to the caecum up to 8 cm proximal to the caecum. It was immediately put into a beaker containing warm Krebs at 35.5 ± 0.5 °C. The contents were flushed with Krebs solution and then placed in an illuminated organ bath, continuously perfused with Krebs at 35.5 ± 0.5 °C constantly bubbled with carbogen gas (95% O_2_/5% CO_2_). The ileum was anchored with pins at the mesenteries of the proximal and distal ends to prevent over-manipulating the tissue. The remaining mesenteries were gently trimmed. The ileum was allowed to acclimatize for 5 min before the recordings were made. Typically, it took ≤10 min from excising the ileum before the recordings were started.

### Bacterial translocation

Bacterial translocation was determined by culturing mesenteric lymph node (MLN), spleen and liver. Mice were administered DOX for 3 months to induce the expression of Tat followed by a period of 2 weeks without DOX to allow for recolonization of gut microbiota. After euthanizing the mice, MLN, spleen and liver were aseptically collected and weighed separately. They were then placed in separate grinding tubes and homogenized by adding 9 parts of sterile-PBS containing 0.1% tween 20 per gram of tissue (e.g. to 0.5 g of MLN add 4.5 mL of PBS containing 0.1% tween 20). 100 μL of homogenate was plated in LB agar. Plates were cultured for 48 h under aerobic conditions. Colonization is quantified as the number of colony-forming units (CFUs) per milliliter of homogenate.

### Data analysis

Results are presented as mean ± SEM. Statistical tests were performed using GraphPad Prism 5.0 software. Analyses of variances (ANOVA) were performed followed by Bonferroni post hoc test when appropriate, or two-tailed paired or Student’s t-test. Values of *P* < 0.05 were regarded as significant.

## Additional Information

**How to cite this article**: Guedia, J. *et al*. HIV-1 Tat exacerbates lipopolysaccharide-induced cytokine release via TLR4 signaling in the enteric nervous system. *Sci. Rep.* **6**, 31203; doi: 10.1038/srep31203 (2016).

## Supplementary Material

Supplementary Information

Supplementary Video 1

Supplementary Video 2
