# Supplementary material for: HIV-1 Tat exacerbates lipopolysaccharide-induced cytokine release via TLR4 signaling in the enteric nervous system
Source: Sci Rep. 2016 Aug 5;6:31203. doi: 10.1038/srep31203 (PMC4974559; doi:10.1038/srep31203)
Supplement: Supplementary Information [file srep31203-s1.pdf]

SUPPLEMENTARY FIGURES AND TABLES

HIV-1 Tat exacerbates lipopolysaccharide-induced cytokine release via TLR4 signaling in the enteric nervous system.

Joy Guedia, Paola Brun, Sukhada Bhave , Sylvia Fitting, Minho Kang, William L. Dewey, Kurt F. Hauser, Hamid I. Akbarali.

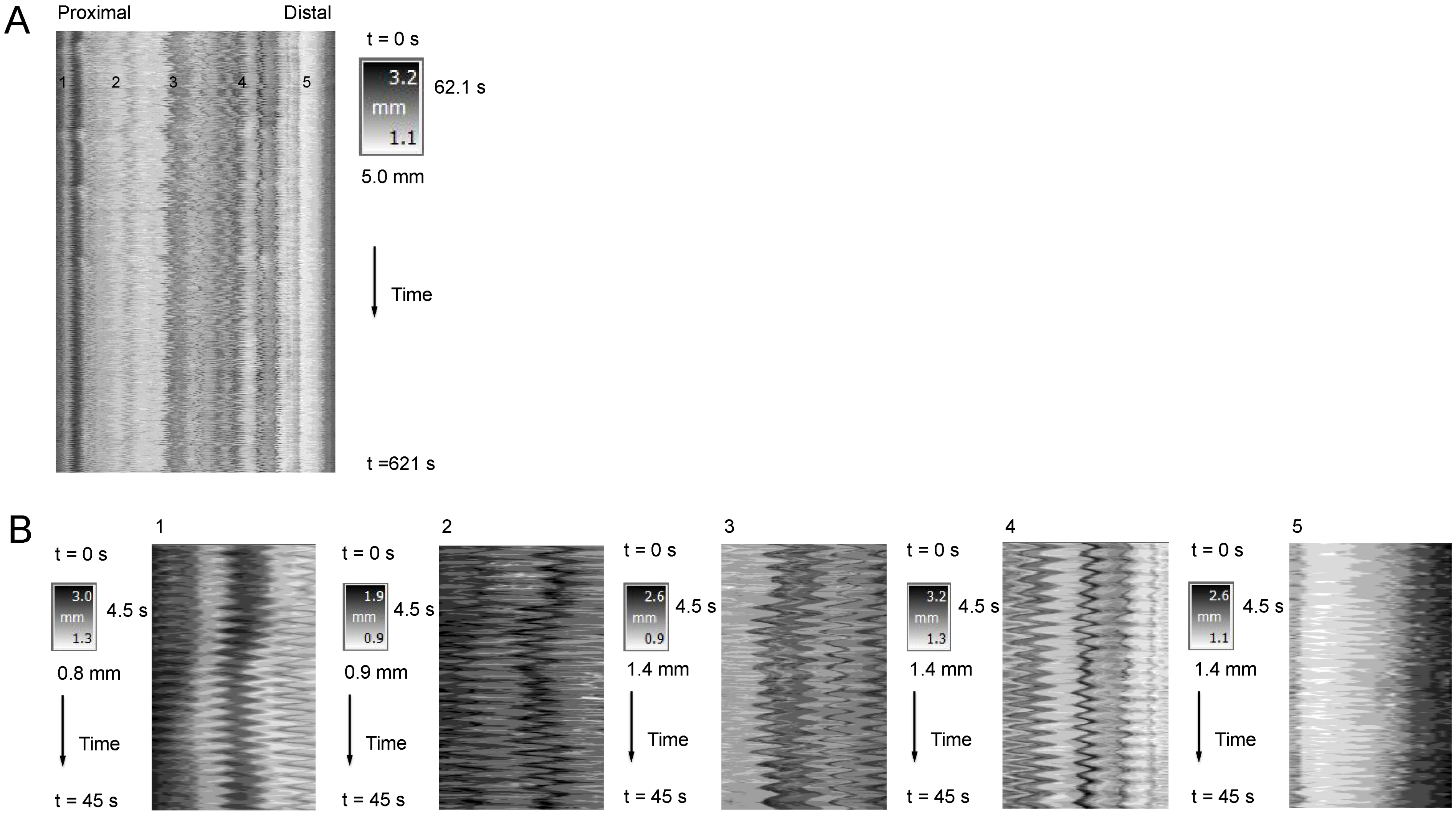

Supplementary Fig 1: Spatiotemporal maps in mouse ileum

A) A representative spatiotemporal map of mouse ileum showing contraction and relaxation of mouse ileum. B) Spatiotemporal maps showing different segments of the mouse ileum zoomed in to show various contraction and relaxation patterns in the first 45 sec of recording

Gene	Forward primer	Reverse primer
Rat IL-6	5'-CCT GGAGTT TGT GAA CAA CT-3'	5'-GGA AGT TGG GGT AGG AAG GA-3'
Rat TNF-α	5'-TGT CGA TGC CTG AGT GGA T-3'	5'-AGG GAG GCC TGA GAC ATC TT-3'
Rat 18S	5'-GAT TAA AGT CCT ACG TGA TCT AAG TCC-3'	5'-TTC GTA ACT GGG AGA AAT TGT AAA-3'
Mouse Na_v1.7	5'-GCC TTG TTT CGG CTA ATG AC-3'	5'-TCC CAG AAA TAT CAC CAC GAC-3'
Mouse Na_v1.8	5′ GTG TGC ATG ACC CGA ACT GAT-3	5′-CAA AAC CCT CTT GCC AGT ATCT-3
Mouse 18S	5'-TCA AGA ACG AAA GTC GGA GG-3'	5'-GGA CAT CTA AGG GCA TCA C-3'
Mouse IL-6	5'-CTA AA GTC ACT TTG AGA TCT ACT C-3'	5'-TGT CCC AAC ATT CAT ATT GT-3'
Mouse TNF-α	5'-GTT GTA CCT TGT CTA CTC CC-3'	5'-GTA TAT GGG CTC ATA CCA GG-3'
Mouse IL-1β	5'-GTA CAA GGA GAA CCA AGC AA-3'	5-TGT TGA AGA CAA ACC GTT TT-3'
Rat MyD88	Santa Cruz MyD88 (r)-PR: sc-106986-PR	Santa Cruz MyD88 (r)-PR: sc-106986-PR

Table 1: Primers used for RT-PCR experiments

	n	Length of Colon	# of Pellets present
**Tat-**	5	5.3 ± 0.5	5.0 ± 0.3
**Tat+**	5	4.8 ± 0.4	4.2 ± 0.7
**Tat-/LPS**	6	5.5 ± 0.4	4.5 ± 0.5
**Tat+/LPS**	6	5.6 ± 0.4	4.8 ± 0.4

Table 2. Original length and number of fecal pellets in mice.

Video Tat (-): Recordings showing pellet propulsion in Tat− mouse colon. Recordings were made for 30 min and the number of natural pellets expelled was counted.

Video Tat (+): Recordings showing pellet propulsion in Tat + mouse colon. Recordings were made for 30 min and the number of natural pellets expelled was counted.

The Tat+ colon expelled all natural pellets, while Tat− colon only expelled 1 pellet.
